# Interventions to Increase the Reachability of Migrants in Germany With Health Interview Surveys: Mixed-Mode Feasibility Study

**DOI:** 10.2196/14747

**Published:** 2020-04-15

**Authors:** Marie-Luise Zeisler, Leman Bilgic, Maria Schumann, Annelene Wengler, Johannes Lemcke, Antje Gößwald, Thomas Lampert, Claudia Santos-Hövener, Patrick Schmich

**Affiliations:** 1 Robert Koch Institute Berlin Germany; 2 Charité Berlin Berlin Germany

**Keywords:** transients and migrants, surveys and questionnaires, cross-sectional studies, feasibility studies, multilingualism and health monitoring

## Abstract

**Background:**

Germany is a popular destination for immigrants, and migration has increased in recent years. It is therefore important to collect reliable data on migrants’ health. The Robert Koch Institute, Berlin, Germany, has launched the Improving Health Monitoring in Migrant Populations (IMIRA) project to sustainably integrate migrant populations into health monitoring in Germany.

**Objective:**

One of IMIRA’s objectives is to implement a feasibility study (the IMIRA survey) that focuses on testing various interventions to increase the reachability of migrants with health interview surveys. Possible causes of nonresponse should be identified so as to increase participation in future surveys.

**Methods:**

The survey target populations were Turkish, Polish, Romanian, Syrian, and Croatian migrants, who represent the biggest migrant groups living in Germany. We used probability sampling, using data from the registration offices in 2 states (Berlin and Brandenburg); we randomly selected 9068 persons by nationality in 7 sample points. We applied age (3 categories: 18-44, 45-64, and ≥65 years) and sex strata. Modes and methods used to test their usability were culturally sensitive materials, online questionnaires, telephone interviews, personal contact, and personal interviews, using multilingual materials and interviewers. To evaluate the effectiveness of the interventions, we used an intervention group (group A) and a control group (group B). There were also focus groups with the interviewers to get more information about the participants’ motivation. We used the European Health Interview Survey, with additional instruments on religious affiliation, experience of discrimination, and subjective social status. We evaluated results according to their final contact result (disposition code).

**Results:**

We collected data from January to May 2018 in Berlin and Brandenburg, Germany. The survey had an overall response rate of 15.88% (1190/7494). However, final disposition codes varied greatly with regard to citizenship. In addition to the quantitative results, interviewers reported in the focus groups a “feeling of connectedness” to the participants due to the multilingual interventions. The interviewers were particularly positive about the home visits, because “if you are standing at the front door, you will be let in for sure.”

**Conclusions:**

The IMIRA survey appraised the usability of mixed-mode or mixed-method approaches among migrant groups with a probability sample in 2 German states. When conducting the survey, we were confronted with issues regarding the translation of the questionnaire, as well as the validity of some instruments in the survey languages. A major result was that personal face-to-face contact was the most effective intervention to recruit our participants. We will implement the findings in the upcoming health monitoring study at the Robert Koch Institute.

## Introduction

### Background

According to the Microcensus of German households, a person has a migrant background if he or she, or at least one parent, has no German citizenship at birth [1]. In Germany, this applied to 19.3 million people in 2017, corresponding to 23.6% of the total population. Of these, 9.8 million had German citizenship, and 9.4 million had another citizenship. Nearly two-thirds of persons with a migrant background (PMB) had migrated themselves (first generation), and one-third were born in Germany (subsequent generations) [1]. Migration has increased since the Second World War, for several reasons. Whereas the recruitment of migrant “guest workers” in the 1950s, and the resulting family reunifications in the 1970s, and European Union enlargement in the 2000s were primarily work oriented, numerous conflicts and wars have led to an increase of refugees coming to Germany, especially since the 2000s [2-4]. The group of PMB in Germany can be described as very heterogeneous, due to differences in the phases of influx, migration background, or specific life circumstances. PMB are underrepresented in nationwide health surveys, such as the health monitoring surveys of the Robert Koch Institute (RKI) in Berlin, Germany [5]. Lower response rates among PMB in population-based surveys have been described in many Western countries [6,7]. The reasons for this can be summarized inter alia as (1) inadequate sampling approaches and hence failing to include PMB in surveys; or (2) barriers to the recruitment of PMB due, for example, to language or cultural issues [8]. It is therefore necessary to evaluate various sampling strategies to include PMB adequately in surveys and to design appropriate recruitment efforts that can minimize nonresponse.

There is an increasing need for reliable data on migrants’ health in order to give a more representative picture of the population. As the national public health institute in Germany, the RKI has the task of extending health monitoring by means of the preferably representative integration of PMB into its health and examination studies. In this context, the Improving Health Monitoring in Migrant Populations (IMIRA) project was initiated, of which the IMIRA survey described here is a part.

### Sampling Strategies

Depending on the objectives of a survey and the associated generalizability of the results to the population, different sampling strategies can be applied. The health monitoring of the RKI aims to cover the German population as a whole; thus, only probability-based sampling approaches can be applied as a specific sampling strategy. In probability samples the selected population is sampled randomly; that means, from a statistical standpoint, the probability of inclusion in the probability sample is predictable and thus results can be generalized. This is not the case for sampling strategies where no inclusion probability is known, for example, snowball or convenience samples, in which participants are recruited by other participants [9,10]. Although nonprobability sampling strategies are considered effective in recruiting hard-to-reach populations [8], the results can hardly be generalized. Probability sampling approaches are frequently based on registers, for example, population registers. In the RKI’s monitoring studies, a 2-stage probability sampling design is applied [11-15]. In the first stage, primary sampling units are selected that are representative of German municipalities. In the second stage, target persons are randomly drawn, according to proportional age and sex strata, through the residents registries (*Einwohnermeldeämter*) within these municipalities. Since Germany has a federal administrative system, in contrast to other European countries, no central residents registry exists [4]. Every municipality therefore needs to be contacted separately.

The residents registry captures various characteristics with which to identify PMB, such as citizenship and place of birth [16]. Citizenship is commonly used for the oversampling of persons with another citizenship to compensate for the assumed lower response rates of PMB. This has been applied in the RKI studies Study of the German Health Interview and Examination Survey for Adults [15,17] and Study of the German Health Interview and Examination Survey for Children and Adolescents (KiGGS) [12-14]. Use of the citizenship characteristic excludes PMB who are naturalized or belong to subsequent generations without non-German citizenship. Place of birth would also be a way to identify PMB with German citizenship. Since it is provided as a string variable in the residents registries, it is prone to error and therefore is not suitable for a big sample [16].

Another important component mentioned in the literature is onomastic procedures, which try to identify naturalized PMB or persons of subsequent migrant generations. These procedures use an algorithm to allocate the origin of a person according to their name [18]. The success of the method might be biased; the name algorithm can lead to more wrong allocations in some PMB groups than in others [19]. Onomastic procedures were not used in the process of sampling at RKI, but to allocate bilingual study information [5,20].

The aforementioned sampling strategies can be considered migrant-sensitive sampling approaches for a better representation of PMB in health surveys. The subsequent steps include measures to increase the response rate, which we describe below.

### Measures to Increase the Response Rate of Persons With a Migrant Background

The heterogeneity of PMB requires a highly differentiated approach for increasing their participation rates in surveys [21]. According to findings in contemporary research, and alongside the challenges and limitations in sampling described above, PMB also have a higher participation threshold than persons with no migrant background, which is among other things presumably due to language and cultural barriers [8,21]. In that respect, lacking the ability to understand the survey language, but moreover illiteracy, might prevent survey participation. A lack of interest in survey participation is also reported as a reason for lower participation rates [7,22]. Researchers have found the following possible reasons for the lower response rate of PMB in surveys: a lack of trust in research, a fear that the reported information might be misused, or cultural beliefs preventing participation in surveys that include intimate or sensitive topics [8]. Specific measures should be taken to increase response rates.

The most obvious way to overcome barriers related to language and literacy skills is to offer multilingual survey materials in plain but culturally sensitive language [23,24]. It is also important to consider the mode of questionnaire administration. Self-administered interview modes, such as online or paper-and-pencil questionnaires, can be differentiated from interviewer-administered modes, such as telephone interviews or face-to-face interviews. A self-administered mode such as an online questionnaire can be accessed easily with a mobile phone and might have a positive impact on the participation of younger and more Web-savvy persons [25]. A paper-and-pencil questionnaire can be filled out without further technical devices and may be associated with less effort for the participants. In general, self-administered interview modes are known to be less subject to effects of social desirability or interviewer influence [26,27].

Nonetheless, telephone or face-to-face interviews can facilitate the participation of persons with literacy problems or other issues in understanding surveys [8,26,28]. Current research emphasizes the importance of personal contact with PMB, such as home visits, in order to increase response rates [8,21]. Personal contact makes it possible to overcome barriers based on mistrust or anxiety, and provides more detailed information about the survey’s objectives [8]. A similar cultural background between the interviewer and participant can increase the response rate [21]. Personal contact leads to a more heterogeneous sample composition according to characteristics of social status, health status, and educational level. Especially vulnerable persons, who would or could not participate in the survey without help or further interventions, can be reached at home. In KiGGS, home visits by specifically trained survey staff proved an effective measure for increasing willingness to participate in the survey and doubled the response rate [20].

Self-administered interview modes were preferred in the most recent RKI monitoring surveys because the sampling strategy involved residents registries, from which only the address of the person could be drawn. This means that, for example, telephone numbers were not available for telephone interviews.

In addition to interventions that directly target participants, some indirect measures are also discussed in the literature. One possibility is to involve the gatekeepers of migrant communities, namely persons who can influence survey promotion and attitudes toward survey participation within the communities, to enhance the participation rate [8]. Furthermore, the involvement of gatekeepers should be considered in order to develop specific public relations when addressing the concerns of PMB [24,29].

### Objectives

Our main objective with the IMIRA project was to identify methodological procedures to further identify methods to better reach PMB in health surveys, and to thus to increase the response rate in future health surveys. The IMIRA survey is a feasibility survey. One focus of the IMIRA survey is to test various interventions that take into account possible cultural and language barriers through the use of a mixed-mode design and multilingual administration. The objectives of the feasibility study (the IMIRA survey) can be summarized as follows: (1) to develop and test the feasibility of an adapted survey design, which is the basis of a subsequent nationwide survey in Germany; (2) to improve the response rate of PMB who have been poorly reached so far and who belong to the biggest groups in Germany; and (3) to identify the causes of nonparticipation in order to increase motivation to participate in health surveys of PMB.

## Methods

### Selection of the Target Population

We applied 2 main criteria for the selection of the target population for the IMIRA survey: the size of the PMB group in the German population, which Table 1 [30] shows; and the experience in previous RKI surveys with the respective PMB regarding response and reachability [5,20]. On the basis of these 2 criteria, we included persons with Turkish, Syrian, Romanian, Croatian, and Polish citizenship in the sample for the IMIRA survey. We selected them according to these criteria regardless of whether they had additional German or other citizenship. We thus excluded from the sample naturalized persons, meaning persons who have acquired German citizenship in replacement of or in addition to another citizenship and persons of subsequent migrant generations with only German citizenship.

We received ethics approval for the IMIRA survey on October 30, 2017 from the ethics committee of the Medical University of Charité, Berlin, Germany (EA1/210/17). The study protocol was approved by the Federal Commissioner for Data Protection and Freedom of Information January 3, 2018 (13-401/008#0085).

**Table 1:** Foreign population in Germany according to citizenship in 2017 [30].

Citizenship	Count, n
Afghan	251,640
Bulgarian	310,415
Croatian	367,900
Greek	362,245
Italian	643,065
Polish	866,855
Romanian	622,780
Russian	249,205
Syrian	698,950
Turkish	1,483,515

### Selection of Sample Points and Sample Size

Congruent with the health monitoring studies at the RKI, we used a register-based random sample in the IMIRA survey. The sampling was a 2-staged procedure: in the first stage, we applied a criteria-based selection of sample points in Berlin and Brandenburg; in the second stage, we randomly selected people according to their citizenship.

#### Step 1: Criteria-Based Selection of Sample Points

We decided to focus on 2 German federal states in the IMIRA survey, with Berlin representing urban regions and Brandenburg representing rural regions. We selected the sample points taking into account the highest proportions of persons without German citizenship in the respective federal states, using data from the Statistical Office in Berlin Brandenburg at the municipal level from 2015 [31]. Since the proportions of persons without German citizenship in Berlin and Brandenburg vary greatly (in 2016 Berlin was home to 16.7% non-Germans, and Brandenburg was home to 4.0% [32]), we focused the selection of our sample points on Berlin. We selected 5 urban sample points (Mitte, Neukölln, Charlottenburg-Wilmersdorf, Friedrichshain-Kreuzberg, and Tempelhof-Schöneberg districts) in Berlin and 2 relatively rural sample points (Cottbus and Fürstenwalde/Spree) in Brandenburg, which met the above criteria.

#### Step: 2 Selection of Persons in the Residents Registry

In the second stage of the sampling, we selected people through the residents registration offices of the chosen sample points. The sampling procedure differed in Berlin and Brandenburg, since not all 5 PMB populations were sufficiently represented in all sample points. In Berlin the target population consisted of people with Turkish, Syrian, Romanian, Croatian, and Polish citizenship, whereas only a few people with Turkish, Romanian, and Croatian citizenship lived in the 2 sample points in Brandenburg. We discovered this through a statistical enquiry in the residents registration offices in Cottbus and Fürstenwalde/Spree on May 24, 2017. Accordingly, we selected only people with Polish or Syrian citizenship in the 2 sample points in Brandenburg. We selected the whole sample with a proportional distribution strata of sexes and 3 age groups (18-44 years, 45-64 years, and ≥65 years): we selected exactly the same number of people in each sex and age category in each citizenship due to the experimental nature of the feasibility study. In this sample we tried to include enough participants in each sex and age category for a better interpretation of our results and the comparison of interventions.

**Figure 1:**
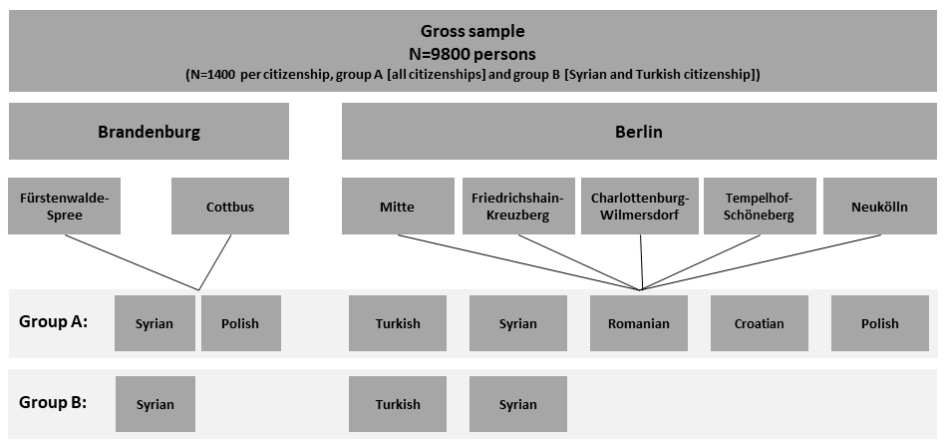
Sample design.

The aim of this survey was to evaluate the effectiveness of specific interventions in increasing response rates. We thus established 2 groups (A and B) for participants with Turkish and Syrian citizenship (the process is detailed in the Data Collection section below). To achieve sufficiently high case numbers in groups A and B of the survey design, we doubled the number of Turkish and Syrian people sampled. We thus selected a total of 1400 people with Romanian, Croatian, and Polish citizenship, and a total of 2800 people of Turkish and Syrian citizenship, resulting in a gross sample of N=9800 for the IMIRA survey (see Figure 1).

#### Questionnaire

The survey instrument was based on the questionnaire of the European Health Interview Survey, which was used and validated in 30 countries [33,34]. Additional instruments included a scale to measure religious affiliation [35,36], an instrument to measure experiences of discrimination [37], and an instrument to measure subjective social status before and after migration [38]. The additional instruments were partly validated in other surveys.

#### Data Collection

##### Survey Design

**Figure 2:**
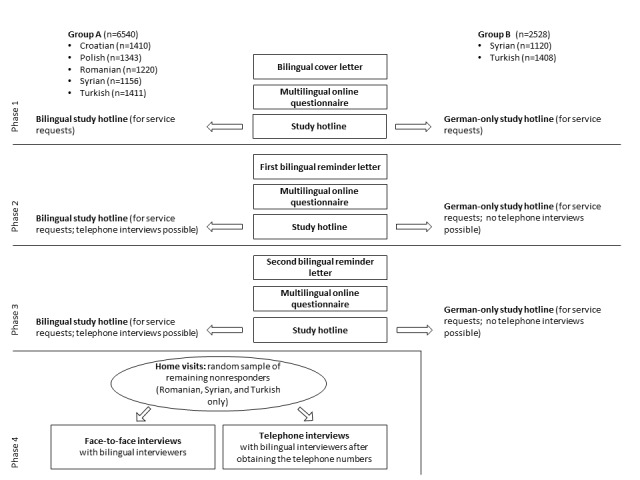
Survey design.

To evaluate the effectiveness of the interventions, we established 2 groups for Turkish and Syrian participants in an experimental design. Whereas group A (intervention group) received further options to participate in the survey, such as face- to-face interviews with bilingual interviewers, group B (reference group) did not (see Figure 2). All participants received a €10 (about US $11) shopping voucher as a conditional incentive. The interventions in group A were presented in a sequential mixed-mode design and are described below.

Qualitative methods such as focus groups with representatives of the migrant populations can be used to identify and evaluate specific measures to attract participants for surveys [24]. The information gained can, for example, be incorporated into the survey materials [22] or help to develop a diversity-sensitive survey design: in addition to the use of multilingual study materials, questionnaires, and interviewers, all materials should use a diversity-sensitive tailored language [21]. To ensure the cultural sensitivity of IMIRA’s cover letters and study materials, we conducted a focus group with representatives from the target populations. The content and design of the cover letter and facilitating factors for survey participation, such as the amount and choice of the incentive, were discussed. Based on the results of the focus group, the cover letters were designed by a professional graphic design company, using pictograms instead of photographs.

##### Phase 1

After we selected the sample in December 2017, we sent the survey information materials by mail to participants in January 2018. These consisted of a cover letter, an information sheet with detailed information on the survey, and a document explaining the data protection measures to the participants. The survey information materials for both groups were in German and in the participant’s language based on their citizenship. All information materials and the questionnaires were translated by a professional translation agency. For the translation, certified translators with native-language level in the required languages were selected by the company. The translators also had experience in translation in the field of health and scientific surveys. The translations were subjected to quality control by the translation agency and subsequent proofreading by a second translator. We collected data for the IMIRA survey from January to May 2018.

The cover letter described the option to participate online using a multilingual computer-assisted Web interview and promoted a toll-free study hotline for queries regarding the survey. The online questionnaire was available in Turkish, Arabic, Romanian, Croatian, Polish, and German in both groups (A and B) of the survey design. To access the computer-assisted Web interview, a personal pincode was necessary. Changing languages during the completion process was possible due to a drop-down language selection menu. This allowed participants to easily switch between the provided languages. The online questionnaire was programmed with Voxco Online software version 5.5.1.205 (Groupe Voxco Inc).

The participants could contact the toll-free study hotline for further information about the survey, to resolve issues with the questionnaire, or to refuse participation. In group A multilingual staff could be contacted through the study hotline. In group B the study hotline was available in German only. Reasons for calling the study hotline were recorded in a process questionnaire, which was also a measure for process standardization and quality assurance. It was programmed with the call-assisted telephone interview software Voxco Command Center version 1.10.5. The process questionnaire recorded queries regarding contact frequency, contact person, result of contact, reasons for nonresponse, and information about the language used during contact. The interviewers also had the option to add further notes about the contact. If the study hotline was contacted to refuse participation, the interviewers were trained to try to convince callers to participate or at least document the reasons for refusal. The interview training took place before the actual start of the survey (field start) in a 2-day intensive training session. On the first day interviewers were informed about the survey objectives and the survey design in detail. In the training participants could address questions to the interviewers, and possible issues and their resolutions were discussed. Interviewers were familiarized with the health interview questionnaire and the process questionnaire that was used to record the hotline calls. On the second day interviewers had the opportunity to practice the use of the software and to click through the questionnaires by themselves. Afterward they practiced possible situations on the study hotline as a role play. During the whole training interviewers could address questions to the project researchers. To remember the training interviewers received a handbook with information about the survey, as well as the most frequently asked questions and problems that might occur. For quick help there was also an overview sheet on the survey with the most important information (Who is conducting the survey and why? What are the survey objectives? Who was invited and how? How can I participate?) at each workstation. Throughout the survey fieldwork the interviewers on the study hotline were supported by experienced supervisors to ensure the quality of data and to provide assistance in difficult situations. The supervisors underwent the same training as the interviewers. In addition, all supervisors had extensive prior experience with scientific surveys and the software used, and were thus able to provide assistance with technical questions. Supervision was also supplemented by project staff who were able to answer questions and problems regarding content.

##### Phase 2

After 2 weeks a reminder was sent to people who had not participated after the first invitation. The reminder promoted the multilingual computer-assisted Web interview again. Participants in group A were given the option to take part in the survey through a call-assisted telephone interview with bilingual interviewers.

##### Phase 3

After 3 weeks a second reminder was sent to participants to promote the survey again, and home visits were offered to group A. Group B participants received a second reminder to complete the online questionnaire. We conducted a focus group with the telephone hotline interviewers after the end of phase 3 to learn about their experiences. The interviewers discussed their personal experience with the IMIRA survey during their work on the study hotline. The focus group was moderated by 1 of the IMIRA survey’s researchers (LB), using an interview guideline. The interview guideline included the following topics: (1) evaluation of the contact design with a focus on reasons for calling the study hotline, mentioned issues or problems, reasons for refusal, and whether these objections could be resolved, and the response to the offer of bilingual interviews; and (2) the interviewers’ opportunity to express their own opinion with a focus on the bilingual materials, study hotline, and translated questionnaire. A log was kept during the focus group by 2 researchers (MLZ) to enable later analysis. We evaluated the log using qualitative content analysis [39]. We formulated categories to analyze the material deductively taking into account the interview guidelines.

##### Phase 4

For the majority of the people sampled (ie, people with Polish or Croatian citizenship or people living in the 2 sample points in Brandenburg), the IMIRA survey ended with the last reminder letter in phase 3. Home visits took place only in a random subsample. The inclusion criteria for the random subsample were as follows: Romanian, Syrian, or Turkish citizenship; allocation to group A; and no response to the further invitation steps. In group B no further interventions were initiated. In this last survey phase we carried out 2 interventions, aiming at a comparison of their effectiveness: (1) home visits for face-to-face interviews using bilingual interviewers, and (2) home visits to obtain the participant’s telephone number to conduct a subsequent call-assisted telephone interview with bilingual interviewers.

The face-to-face interviews were carried out using a tablet with internet access and the online questionnaire. To evaluate the 2 approaches, participants were randomly allocated to the face-to-face interview group or the phone number group. Both interventions were aimed at increasing the response rate further and gaining more information about the target population, for example, reasons for nonresponse, German language skills, or validation of the addresses. An address was not replaced if it was wrong or unavailable. These data were documented in a process questionnaire similar to that used for the study hotline.

Whenever possible the interviewers were accompanied by an experienced supervisor to ensure data quality and reduce uncertainties in handling the tablet. Based on the experience of the first interviewers’ focus group, a second one was carried out at the end of the home visit phase. The interviewers’ experiences enriched the quantitative data gathered with the process questionnaire. The focus group was moderated by an IMIRA researcher (LB) using an interview guideline. The interview guideline was structured in 3 main parts: (1) experiences with the home visits, including situations at the door, experiences during face-to-face interviews or the retrieval of telephone numbers, and reactions to bilingual contact; (2) technical and organizational framework regarding address quality, tablet handling, or operating times; and (3) the interviewer’s personal opinion about the effectiveness of the home visits. A log was kept by 2 researchers (MLZ) for subsequent qualitative content analysis. This was done in a similar way to that in the first interviewers’ focus group.

Phase 4 can be considered a benchmark test for a specific subsample of nonresponders. No evaluation in comparison with group B was possible due to the study design.

## Results

### Response to the Survey

Data collection was completed in May 2018. The response rate was 15.88% (1190/7494) over all target populations, such that 1190 questionnaires were completed by our participants. We published the first results on response rates, the effectiveness of the interventions, and sample composition in 2019 [40].

### Gross Sample

We selected 9800 people, stratified by age and sex, for the gross sample and requested their data from the residents registries in Berlin and Brandenburg. Originally, we aimed at an equal distribution of age groups and sexes within the citizenships for sampling. Our final gross sample consisted of only 9068 people due to the lack of people aged over 65 years in some citizenships. In particular, there were few Syrian and Romanian people aged over 65 years in the lists provided by the residents registries. In Brandenburg this was also problematic for Polish people (see Table 2). The sample thus systematically lacked older age groups.

**Table 2:** Frequency distribution of the gross sample by sample point, citizenship, group, sex, and age.

Characteristic		Sample point^a^														Total (N=9068)
		Berlin (n=8255)										Brandenburg (n=813)				
Citizenship		Turkish		Syrian		Romanian		Croatian		Polish		Syrian			Polish	
Group		A (n=1411)	B^b^ (n=1408)	A (n=905)	B (n=880)		A (n=1220)		A (n=1410)		A (n=1021)		A (n=251)	B (n=240)	A (n=322)	
**Age range (years): male**		706	703	473	463		606		705		511		131	126	155	4579
	18-44	255	255	217	212		300		265		185		68	66	68	1891
	45-64	242	240	151	148		213		217		183		52	50	68	1564
	≥65	209	208	105	103		93		223		143		11	10	19	1124
**Age range (years): female**		705	705	432	417		614		705		510		120	114	167	4489
	18-44	257	259	212	207		301		249		183		68	66	68	1870
	45-64	237	236	159	152		207		227		181		46	44	66	1555
	≥65	211	210	61	58		106		229		146		6	4	33	1064

^a^In Berlin it was possible to draw people with all 5 target citizenships from the residents registry. In Brandenburg this was not practicable, and only people with Syrian and Polish citizenship were selected due to low numbers of people with Turkish, Romanian, and Croatian citizenship.

^b^Reference group B was drawn from Turkish and Syrian citizens only.

### Final Disposition Codes

At the end of the survey, we assigned every sampled case a final disposition code to enable the later analysis of response rates. We adapted the disposition codes from the guidelines of the American Association for Public Opinion Research (AAPOR), differentiating the sample into 4 categories: (1) interviews (ie, cases with a completed questionnaire); (2) refusals (R), noncontacts (NC), or other (O) (ie, cases we had contact with, not resulting in participation, eg, people who called the study hotline to refuse participation; (3) cases of unknown eligibility (UE) (ie, cases we did not know anything about, eg, people who did not react to our contact attempts at all; and (4) cases that were not eligible (NE) for the survey (ie, the selected person was unknown in the household or the address was not correct, eg, when mailed cover letters were returned as undeliverable or we received information that someone had died) [41].

**Figure 3:**
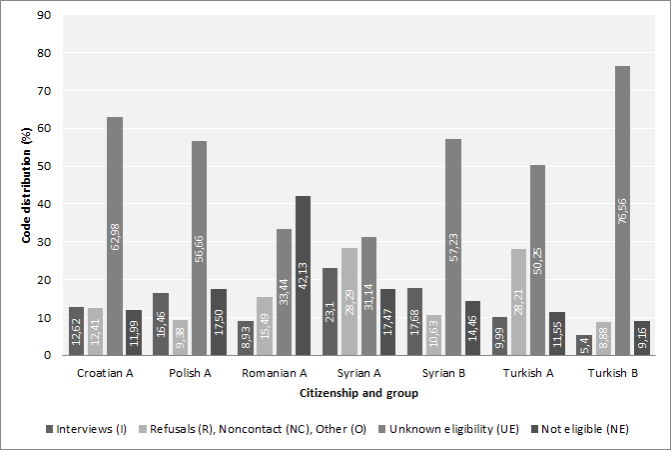
Distribution of American Association for Public Opinion Research disposition codes (%) by citizenship and assignment to intervention group A or control group B.

As Figure 3 shows, the distribution of the disposition codes varied greatly with regard to citizenship. Most completed interviews were conducted with Syrian (group A: 267/1156, 23.10%; group B: 198/1120, 17.68%), Polish (221/1343, 16.46%), and Croatian (178/1410, 12.62%) participants. Significantly lower proportions of Turkish (group A: 141/1411, 9.99%; group B: 76/1408, 5.40%) and Romanian (109/122, 8.93%) participants than participants with other citizenships completed the interviews. Contact resulting in no participation (R NC O) were most frequent with the Syrian (327/1156, 28.29%) and Turkish (398/1411, 28.21%) participants of group A, followed by Romanian (189/1220, 15.49%) and Croatian (175/1410, 12.41%) participants. All other citizenship groups had R NC O shares of around 10% (Polish group: 126/1343, 9.38%; Syrian group B: 119/1120, 10.63%; Turkish group B: 125/1408, 8.88%). Differences between the citizenships and the survey design for groups A and B can be identified regarding cases with no further information about eligibility (UE). UE had the greatest share in the Turkish group B (1078/1408, 76.56%), Croatian (888/1410, 62.98%), Syrian group B (641/1120, 57.23%), and Polish (761/1343, 56.66%) participants. Among Romanian participants, only 33.44% (408/1220) were UE, in contrast to 42.13% (514/1220) of the cases that were NE. The percentage of Romanian NE cases was 3 times higher than for the other citizenships, with only 13.50% (1060/7848) of NE cases on average, ranging from 9.16% (129/1408) in the Turkish group B to 17.47% (202/1156) in the Syrian and 17.50% (235/1343) in the Polish group A. When comparing the intervention group A with the control group B, it was striking how the measures in group A led to an increase in participation and, furthermore, to more information about the sample.

### Process and Qualitative Data From the Study Hotline

#### General Results

The study hotline was available during the first 3 survey phases from January to April 2018. Outside the interviewers’ working hours an answering machine was set up. We received 409 contacts during this period; 21 of these were repeat contacts. Most calls came from Syrian (130/405, 32.1%), Turkish (101/405, 24.9%), and Croatian (83/405, 20.5%) participants. There were no significant differences between the contact frequency of group A (multilingual hotline; 296/6540, 4.52%) and group B (German hotline; 109/2528, 4.31%). The study hotline was evaluated positively in the interviewers’ focus group. Participants who had been living in Germany for a long time associated a “warm and nostalgic feeling” with the multilingual hotline. The interviewers described the use of the native language as an opportunity “to get a foot in the door.” Personal stories, hobbies, or the same origin facilitated the communication process and built “a feeling of connectedness,” Polish, Romanian, and Croatian interviewers reported that the language changed frequently during a single call. The Syrian interviewers reported the use of the Arabic language in most cases, but they faced great difficulties due to the variety of Arabic dialects. Similar experiences were reported by Turkish interviewers, who claimed that communication was impossible with Kurdish-speaking people. Communication in group B was not entirely possible due to the lack of bilingual interviewers.

Call times varied between 5 and 41 minutes in both groups, excluding calls during which telephone interviews took place. The hotline was contacted slightly more often by women (198/384, 51.6%) than by men (186/384, 48.4%), which holds true for all citizenships except Syrian, where more men called the study hotline. In general the hotline was most often used by elderly people. In the Croatian, Polish, and Romanian groups, the share of elderly people (≥65 years) was more than 50% (96/168, 57.1%). In the Syrian group there was no difference in contact frequency by age. The interviewers reported that loneliness and the ability to speak to someone were relevant factors in calling the study hotline for older respondents. A total of 70.7% (289/409) of contacts were established by the target person (the person invited to participate in the IMIRA survey). The only exception was in the Turkish group, where only 58.4% (59/101) of contact was with the target person. When the call was made through a contact person, it was mainly a close family member, such as a child or partner. The reasons for calling the study hotline were documented in a process questionnaire in 4 categories: (1) queries regarding the survey, (2) issues regarding participation, (3) telephone interview (possible from phase 2), and (4) refusal.

#### Queries

Of all contacts through the study hotline, 23.6% (107/453) involved general queries about the survey. The most queries reported by the interviewers in the focus group involved participation (“Do I need to go somewhere?”), content of the survey (“Why is the survey being conducted?”), and the contracting authority of the survey (“Who is conducting the survey?”). There were slightly fewer questions about the sampling of respondents (“Why was I chosen to participate in the survey?”) and data protection (“Will my data be shared with others?”). Data from the process questionnaire provided further information about the citizenship of the callers. Syrian people mostly had queries regarding participation, the content of the survey, and data protection. Questions about the contracting authority or sampling were more often asked by Croatian and Polish people.

#### Issues

Issues with participation were the reason for calling the study hotline in 9.7% (44/453) of contacts. The problems documented in the process questionnaire were largely technical: the online questionnaire could not be opened, the code to participate in the survey was not working, or there was no internet access or device available for online participation. Some callers requested a paper questionnaire. These technical issues were more often reported by Polish, Croatian, and Syrian participants. The Syrian interviewers reported that literacy problems and illiteracy made it impossible for some participants to take part in the survey without a telephone interview.

#### Telephone Interview

A telephone interview was promoted in the first reminder in group A, resulting in 93 telephone interviews lasting an average of 50.5 minutes. Of all the callers, 9.3% (42/453) asked directly for the telephone interview as promoted in the cover letters. Participation via telephone interview varied greatly between the citizenships. Most telephone interviews took place in the Croatian (n=29), Polish (n=25), and Syrian (n=22) groups, while Romanian (n=9) and Turkish (n=8) callers did not use this option.

Issues with the telephone interview were reported in the interviewers’ focus group, which could not be found in the descriptive data of the process questionnaire. According to the interviewer, the quality of the translated questionnaires was not very good. Although the questionnaire was translated by a professional translation agency, incorrect translations were belatedly identified in the process of data collection. The questionnaire was also perceived as very long and complex, and therefore unsuitable for call-assisted administration. According to the interviewers, participants rated the questionnaire as very intimate and “psychologically stressful,” especially if they had serious diseases or traumatic experiences that affected their health status permanently. Interviewers were thus forced into the role of a psychological therapist when participants told them about their traumatic experiences due to war, torture, or violence. In these cases contact information was provided for advisory offices specializing in these issues, to lessen the burden on the interviewers. Specific problems were also identified, such as questions regarding religion, which were considered offensive in the Syrian and Turkish groups, or sociodemographic questions about health, which were considered to be irrelevant in the Romanian group.

#### Refusals

Half the callers intended to refuse participation (237/453, 52.3%). They were predominantly Turkish, Syrian, or Croatian citizens. Even though interviewers were trained to promote the survey, the majority could not be persuaded.

The most frequent reason for refusal was a lack of interest in the survey (148/384, 38.5%). Interviewers in the focus group reported that they tried to convince people who commented on the topic of the survey that it was “none of my business.” Polish interviewers referred to different living environments and explained that Polish people were predominantly in Germany for work, not to live permanently. Thus, questions about health were not of interest because health-related action, such as visits to the doctor, took place in Poland rather than in Germany. The 2 reminder letters, and especially the announcement of a home visit as part of the last reminder, were perceived as “nuisance,” “invasion of privacy,” and “involuntary.” According to the interviewers, the objectives of the survey were not well understood. Participants reported having “no trust that something will change due to their participation” and criticized the focus on PMB (“Are there diseases that only affect special population groups?”). The interviewers reported that some callers complained about being assigned to the group of migrants and felt discriminated against in the survey. Croatian, Syrian, and Turkish participants most commonly refused due to having “no interest.”

The second most frequent reason for refusal was a lack of time (57/384, 14.8%), which was particularly common for Croatian, Syrian, and Turkish participants. Other reasons that prevented participation were health restrictions or literacy issues, predominantly given by Syrian and Turkish participants. The interviewers reported that there were various calls reporting that participants had moved to another address or were deceased. Although these interviewers reports could not be confirmed quantitatively, confrontation with death was considered challenging by the interviewers and was highlighted in the focus group.

### Process and Qualitative Data of the Home visits

#### General Results

Of the 1822 participants selected for the home visits, only 945 (51.87%) could be visited by the Romanian, Syrian, and Turkish interviewers in the given time. Overall the interviewers reported that contact with target people was “mostly polite and friendly.” The Turkish interviewers in particular evaluated their strategies of persuasion in direct personal contact with the sample targets as particularly convincing. Romanian interviewers emphasized the positive effect of the home visits with the following statement: “If you are standing at the front door, you will be let in for sure,” meaning that the main challenge was not convincing people to participate, but making first contact. When personal contact was accomplished, it was with the target person directly in 78.5% (551/702) of cases. Of these contacts with the target persons, 58.9% (334/567) led to survey participation.

Differences could be identified between the groups: Syrians consented in 74.4% (203/273) of all cases, whereas Turkish participants did so in only 43.7% (101/231). This is in line with the overall response rate to the survey. More Turkish participants than people with other citizenships also needed to be contacted more than once. The contact language was mainly not German. Interviewers reported that it was important for some participants to be able to use their mother tongue during the interview. This was especially demonstrated by the fact that no interviews in the last survey phase were conducted in German, although interviewers estimated that German was possible in 9.7% (45/465) of communication. Interviewers reported different characteristics of language as related to citizenship. Older Turkish participants were more frequently not German speaking; thus, bilingual approaches should be taken into account for this group. Romanian participants could not be contacted in German during the home visits at all. Similar experiences were reported by the Syrian interviewers, where communication took place in Arabic only. They stated that some medical terms were not understood in Arabic but were understood in English. Language barriers that could not be attributed to the questionnaire itself were reported for people of all citizenships, for example, challenges with Kurdish-speaking people in the Turkish or Syrian group or Russian-speaking people in the Romanian group.

#### Interviews

Of all contacts, 11.0% (104/945) led to a face-to-face interview performed in the participant’s household directly, and 5.5% (52/945) led to a subsequent telephone interview after the telephone number was recorded (100/945, 10.6%). The interviewers’ focus group noted that obtaining the phone number for a subsequent telephone interview should be considered an emergency option if the respondent desired no alternative. Both participation options during the fourth survey phase were taken up by Syrian or Turkish participants. Of the participants targeted, 51.2% (334/652) were female. Slightly more often women participated in face-to-face interviews (57/100, 57.0%) than in the telephone interviews (25/46, 56%), which had a more balanced sex distribution. Participants were generally 18 to 64 years old (114/146, 78.1%). The youngest age group (18-44 years) was mainly represented by Romanian (14/58, 24%) and Syrian participants (31/58, 53%), and more people from the older age group (≥65) participated from the Turkish group (18/32, 56%) (see Table 3).

**Table 3:** Age and citizenship frequencies in the interviews in the home visit phase.

Citizenship	Age range (years)			Total (N=146)
	18-44 (n=58)	45-64 (n=56)	≥65 (n=32)	
Romanian	14 (24.1)	8 (14.3)	2 (6.3)	24 (16.4)
Syrian	31 (53.4)	25 (44.6)	12 (37.5)	68 (46.6)
Turkish	13 (22.4)	23 (41.1)	18 (56.3)	54 (37.0)

The interviewers described various situations in which they felt stressed, for example, when they were confronted with diseases, misfortunes, or participants “tending to show depressive symptoms.” In these situations they stated that it was difficult to keep their distance or interrupt the flow of speech without being offensive. The traumatic experiences of some participants (eg, during war) led to a feeling of helplessness for the interviewers. In this respect the questionnaire was described as very formal, as opposed to the personal character of the home visits. Similar to the experiences in the study hotline, the interviewers reported that questions regarding migration history or religious affiliation offended some participants so much that interviewers had difficulties continuing the interview. Questions regarding income or the consumption of alcohol were also viewed critically.

#### Refusals

Of all contacts during phase 4, 33.2% (314/945) resulted in refusal, especially in the groups of Syrian (173/314, 54.8%), Turkish (76/314, 24.2%), and Romanian (42/314, 13.4%) participants. The main reason for refusing participation was “no interest” in the survey (138/235, 58.7%). Interviewers stated that there were some issues involving the RKI that led to a misunderstanding about the survey’s aims or the importance of research in general. Interviewers were, for example, forced to explain the RKI and its function because it was not known by the respondents. In the Turkish and Romanian groups, skepticism about and mistrust in research caused refusal. There was, for example, no understanding of data security and data sharing. Some participants felt as if they were under surveillance by the German government and believed that hidden information was being gathered with the survey. As in the process of standardization for the study hotline, interviewers were asked to evaluate the effectiveness of their attempts to convince participants after refusal. The interviewers were not successful in handling objections in 96.1% (172/179) of all refusals. Syrian participants were most likely to be convinced, but only half participated in the end, which was the same finding as in the study hotline.

#### Sample Dropouts

A total of 26.8% (253/945) of the participants could not be reached during the home visit phase. More than half of these sample dropouts (138/253, 54.2%) were Romanian participants, confirming the previous results shown for the AAPOR disposition codes. The reasons for sample dropout were very different between populations. Whereas Romanian participants were mainly not available due to wrong addresses or the lack of a name on the front door or mailbox, it was predominantly relocations that caused nonparticipation in the Syrian group. Interviewers also reported that they applied different strategies to overcome these barriers; for example, 1 of the Romanian interviewers walked up the staircase in the residential houses to identify the right apartment if no name was on the doorbell nameplate. Syrian interviewers reported that sometimes there was no possibility of reaching participants living in shared accommodation, since no name was on the doorbell or because access was blocked by security guards. The interviewers thus evaluated the reachability of participants and quality of addresses very differently.

## Discussion

### Principal Findings

The main objective of the IMIRA survey was to identify recruitment measures to improve the response of PMB to health surveys by the RKI. Although the IMIRA survey focused on people with non-German citizenship only, the findings are of value for designing subsequent health surveys at the RKI. We tested multiple interventions in the IMIRA survey, which focused primarily on overcoming language barriers, enhancing personal contact with bilingual interviewers, and optimizing bilingual survey information materials or a multilingual questionnaire. The emphasis of the IMIRA survey was on diversity-sensitive information with a special focus on language and the design of the documents. The materials consisted of a cover letter and a survey brochure, and were developed with representatives of the target population in a focus group. Although the lack of varying materials for different citizenships was criticized in the focus group, this idea of varying materials was waived due to limitations to the capacity of survey management. Our experience with this survey shows that alterations for people with different citizenships should be implemented, although it might lead to additional costs. Otherwise, there is a risk that participants would feel discriminated against or misunderstand the survey’s objectives, as happened in this IMIRA survey. The main reason for this was that the survey materials were not tailored for each citizenship group. Germans with additional foreign citizenship and people with only foreign citizenship were not differentiated in the IMIRA survey. Addressing the whole sample as migrants resulted in an external attribution that might have been in contrast to people’s own personal attribution of origin or affiliation, and this might lead to a feeling of discrimination. This aspect has been discussed in recent research and should be taken seriously when designing survey materials or a communication strategy in subsequent surveys [42].

Personal contact is very often described in the literature as the “silver bullet” for achieving better response rates in hard-to-reach populations [20,21,28,43]. For PMB, who can also be considered hard to reach, personal contact was an essential component of the IMIRA survey design. The study hotline was set up with bilingual interviewers offering the opportunity to address queries regarding the survey or participation directly. Home visits with bilingual interviewers were carried out in the last survey phase, turning the previously rather indirect contact into a direct contact. The interviewers were members of the target population themselves and thus capable of addressing the survey topics not only in the relevant language, but also on a diversity-sensitive basis. This was intended to minimize inhibitory factors, such as lack of trust in research or literacy issues, which were often discussed in the literature, or a lack of internet access [8,21]. The attempt to eliminate language barriers worked very well, as the findings show. Interviewers reported the frequent use of languages other than German on the study hotline. Interviewers on the German-only hotline (group B) in particular reported that sometimes no communication was possible due to language issues. Communication during the home visits took place almost exclusively in languages other than German and would not have been possible in German according to interviewer assessments. Home visits increased participation frequency. Personal contact through the telephone was not as effective as we had assumed it would be and had no effect on response. The study hotline was primarily used for refusals and less for queries regarding the survey, and thus primarily had an informative value.

Another important emphasis of the IMIRA survey was on offering different participation options and interventions to lessen the burden of participation. Our respondents had the option to participate online, by telephone, or (only in a small subsample) face-to-face, using multilingual questionnaires and bilingual interviewers to overcome language barriers as recommended in research [21,22]. The questionnaire was administered in a mixed-mode design, known to be connected to higher response rates, since it takes a variety of participation preferences into account [44]. Participants were first invited to participate online. With each new contact, other modes, such as telephone or face-to-face interviews, were offered sequentially in group A. This did increase the response rate and in previous research was shown to be more effective than a simultaneous mixed-mode design [45]. In consideration of the heterogeneity of the target population, a combination of self- and interviewer-administered questionnaires seemed to be of value to facilitate survey participation [46], although it might have had implications for data quality in terms of, for example, interviewer effects or the discrepancy between auditory and visual comprehension of the questionnaire [47,48]. The online questionnaire was accessible via mobile phone: participants had the opportunity to answer the questionnaire “on the go.” This was intended to reduce refusals for time management reasons. The questionnaire could also be interrupted, further promoting compatibility with the participant’s leisure behavior. Research has shown that younger people might be more attracted by the opportunity to participate online, whereas older people tend to prefer interviewer-administered modes (telephone or face-to-face interviews) [49]. This was confirmed in the IMIRA survey with regard to the use of the study hotline and home visits, where the composition of the sociodemographic sample differed significantly between self- and interviewer-administered modes.

Alongside the survey’s objective to increase responses, another important aim was to identify reasons for nonresponse of PMB. Every contact with the participants, through the study hotline or through the home visits, was therefore documented in a process questionnaire. The results showed that more than half of the callers wanted to refuse participation. Interviewers were required to document the reasons as accurately as possible. We identified differences between citizenship groups: Croatian, Syrian, and Turkish participants primarily called the study hotline to refuse participation. During home visits Turkish and Syrian participants more frequently refused participation. The most common reasons in both cases were “no interest in the survey” or “no time.” Interviewers in the focus groups described the refusal reasons in more detail than was possible in the process questionnaire. They stated that participants felt disturbed by the survey, did not understand the survey’s aims, or had no interest in the research and the possible implications of the survey results in their everyday life. This is similar to the increasing trend of survey fatigue found in other research in recent decades [50,51]. More frequent headlines in the press concerning the falsification of survey results could also explain this negative trend. Other reasons for refusal were given by the interviewers as well, involving the subject of the IMIRA survey. Although this could not be confirmed quantitatively, interviewers described the survey’s focus on improving health monitoring in migrant populations as causing conflict, especially for those who did not identify with the label “migrant.” According to the interviewers, there was suspicion that people who had lived for a long time in Germany were being put on a level with recent refugees or newly migrated people. Complaints about this were predominantly made by Croatian and Romanian participants, whose migration histories are from a more distant past. The Turkish interviewers described other issues regarding the focus on migrants. Many participants did not react to the survey invitations. Only during home visits could participants be questioned about their unwillingness to participate. The reported indifference to survey participation might be the result of being ignored by German institutions and politics for a long time and a resulting development of social segregation from the host population [24,52]. Interviewers stated that the only way to convince Turkish people to participate was to argue at the institutional level, for example, emphasizing that the government was now interested in their health status and that it was therefore important to provide the information.

In addition to providing detailed reasons for refusal, the process questionnaire also improved the quality of the sample with regard to dropouts due to relocation, death, or wrong addresses. This was striking especially in the Romanian group, who had the greatest share of noncontactable (NE) people in the whole sample. A large proportion of these cases were identified by undeliverable mail; their address was wrong or nonexistent. Other cases could only be identified by the interviewers in the home visits phase, especially when no name was on the doorbell or mailbox, or if the apartment was sublet. The high mobility of people with Romanian citizenship was in contrast to the relatively rigid system of population registration, which makes the sampling of mobile groups more difficult and does not seem appropriately representative [16,53].

### Limitations and Challenges

We were bound by the general procedure at RKI when designing the IMIRA survey, using population registers for sampling. Two challenges became clear as a result. First, we had to sample people according to their citizenship, since there was no other practicable way to sample PMB for our survey, as described earlier in the theoretical considerations. As a result we excluded naturalized PMB and subsequent generations of PMB from our target population, who had no citizenship other than German. Second, the sample size decreased more than expected, beginning with insufficient numbers of people who could have been selected for the sample. We requested a disproportionate sample from the residents registration offices, stratified by sex and age for each citizenship. Each cell should have been filled equally for a better comparison of the groups in later analysis. The sample could not be delivered as requested, especially for the Syrian and Romanian groups 65 years of age or older, and for the 2 sample points in Brandenburg for all citizenships in the oldest age category. Of 9800 requested individuals, a sample of only 9068 people was delivered. The sample size was also decreased by invalid addresses due to relocation or for other reasons. In previous RKI studies, the quality-neutral dropout rate due to invalid postal addresses was about 10% to 15% of the gross sample [12]. According to research it can be assumed that the percentage may be even higher in mobile population groups, such as newly arrived migrants or asylum seekers [16]. The quality-neutral dropout rate in the IMIRA survey was 17.36% (1574/9068), with great differences between the citizenships, ranging from 10.74% (169/1574) in the Croatian group to 32.66% (514/1574) in the Romanian group. There were a variety of reasons for the reduced gross sample, and these should be considered when drawing a sample from population registers. In future surveys it would be advisable to use a proportional stratified sample of the target populations. In addition, the possible dropout rate should be taken into account and the gross sample should be increased accordingly. For some target populations this might still be an inadequate sampling method, and other ways of sampling should be taken into account.

Although the survey design was sequential, the effectiveness of the various interventions cannot clearly be separated, since the prior interventions were still accessible when the new interventions were initiated. We also used the survey’s experimental approach to evaluating the interventions only for Syrian and Turkish participants and for only the first 3 survey phases. The effectiveness of the interventions could not be evaluated for the other citizenships, and the analysis was descriptive only. The impact of home visits on response rate could be evaluated only for Romanian, Syrian, and Turkish participants.

The questionnaire was available to all participants in Arabic, Croatian, German, Polish, Romanian, Syrian, and Turkish. According to the interviewers, who were native speakers, the translation of the questionnaire was not entirely adequate. In addition, the questionnaire was not culturally adapted for the target populations. As described in the results, some interviewers reported that respondents had strong reservations about certain questions, for example, whether a respondent was a member of a religious community. The negative experiences with the quality of the translation will have consequences for future surveys. We will conduct a cognitive test of instruments that are not already validated in the requested languages and that might be identified as particularly sensitive with representatives of the target populations. We are endeavoring to develop standard operating procedures for externally commissioned translations at the RKI to ensure the quality of future studies. We will use the team approach for translations, in which 2 independent translators simultaneously produce a translation. Differences between the 2 translations are discussed by the team, meaning between the 2 translators and a moderator who acts as a mediator [54].

Further studies in this field of research should acknowledge these limitations. In particular, the evaluation of various aspects of the survey recruitment measures should be studied in more detail. For example, to our knowledge, there is still no research into the effect of multilingual questionnaires on response rate. Experimental studies are thus needed.

### Conclusion

The IMIRA survey met the challenges of low response rates of PMB with tailored interventions, which were shown to be more or less effective. Bilingual information material and questionnaires were received very positively by the participants, although there were some issues regarding translation that we hope to avoid in future surveys through the team approach. The study hotline did not have the expected effect, but it proved to be of value for participants with queries, with technical or other survey-related issues, or wanting to refuse participation. It is therefore advisable to establish a contact channel for respondents in future surveys. The study hotline also enabled us to draw lessons for the future in terms of structuring survey information materials and providing the required support for participants. Home visits with bilingual interviewers had the greatest effect on responses and will be a firm component of our future surveys when considering PMB. We gathered qualitative and procedural data through focus groups with the interviewers and contact protocols during the fieldwork phases, providing information about reasons for refusal or issues leading to nonresponse, and helping to better characterize the sample in general. Nevertheless, some of the mentioned reasons for nonresponse were still very unspecific and should be investigated in more detail in subsequent surveys.

## Abbreviations

AAPOR: American Association for Public Opinion Research
IMIRA: Improving Health Monitoring in Migrant Populations
KiGGS: Study of the German Health Interview and Examination Survey for Children and Adolescents
NC: noncontacts
NE: not eligible
O: other
PMB: persons with a migrant background
R: refusals
RKI: Robert Koch Institute
UE: unknown eligibility
